# Challenges in linking interferon signatures to clinical trajectories in juvenile idiopathic arthritis

**DOI:** 10.1186/s12969-026-01256-x

**Published:** 2026-08-18

**Authors:** Laura Scagnellato, Melissa Kartawinata, Wei-Yu Lin, Stephanie Shoop-Worrall, Roberta Ramonda, Chris Wallace, Lucy R. Wedderburn

**Affiliations:** 1 Infection, Immunity and Inflammation Research and Teaching Department, University College London (UCL) Great Ormond Street Institute of Child Health, London, UK; 2 National Institute for Health and Care Research (NIHR) Biomedical Research Centre at Great Ormond Street Hospital, London, UK; 3 Rheumatology Unit, University Hospital of Padova, Padova, Italy; 4 Centre for Adolescent Rheumatology at University College London, University College London Hospital and Great Ormond Street Hospital, London, UK; 5 Medical Research Council (MRC) Biostatistics Unit, University of Cambridge, Cambridge, UK; 6 Centre for Epidemiology, University of Manchester, Manchester, UK; 7 Centre for Health Informatics, The University of Manchester, Manchester, UK; 8 Cambridge Institute of Therapeutic Immunology and Infectious Disease, University of Cambridge, Cambridge, UK; 9 Department of Medicine, University of Cambridge, Cambridge, UK

**Keywords:** Biomarkers, Juvenile idiopathic arthritis, Methotrexate, Response, Artificial intelligence

## Abstract

**Background:**

Based on the recent finding that higher baseline expression of interferon (IFN)-driven gene signature in blood positively correlates with good response to methotrexate (MTX) in Juvenile Idiopathic Arthritis (JIA), this study tested the hypothesis that baseline IFN scores differ significantly between trajectory groups of response to MTX, defined by machine learning models enabling clinicians to predict not just the likelihood, but the specific longitudinal course of therapeutic response.

**Findings:**

Longitudinal clinical data from 699 children and young people (CYP) with active JIA initiating MTX and a nested sub-cohort of 102 CYP with previously available baseline blood transcriptomic data were analysed. Clinical disease courses were classified using multivariate group-based trajectory modelling, while baseline interferon activity was quantified using established 51-gene and 5-gene expression signatures derived from bulk RNASeq. There was no association between pre-treatment 51-gene and 5-gene IFN response scores and distinct clinical disease trajectories following methotrexate initiation by regression modelling. Post-hoc calculations indicated that the study was underpowered (44–58%) to detect medium effects, demonstrating that the increased clinical resolution provided by trajectory analysis significantly compromises statistical power in size-limited paediatric cohorts.

**Conclusions:**

Clinical trajectories accurately describe patient heterogeneity, but the IFN score does not represent a marker for a specific trajectory group?. Future stratification strategies require larger multi-centre cohorts to identify non-responders early and exploit the window of biological opportunity.

**Supplementary Information:**

The online version contains supplementary material available at https://doi.org/10.1186/s12969-026-01256-x.

## Background

Juvenile Idiopathic Arthritis (JIA) is an heterogenous condition affecting children and young people (CYP), whose disease activity is often prolonged into the adult age [1, 2]. The management of JIA has evolved significantly over the past decades, driven by the widespread use of disease-modifying anti-rheumatic drugs (DMARDs) and biologic agents. Although the prognosis has improved significantly with the advent of new drugs, methotrexate (MTX) remains the cornerstone and first-line treatment for most JIA subtypes [3]. However, the response to MTX is heterogeneous and tolerability is often an issue for children and caregivers [4, 5]. We have shown that clinical outcomes may follow complex trajectories, sometimes characterised by discordance between physician assessments and patient- or parent-reported measures [6, 7]. These measures capture different aspects of the disease and of the patient’s journey and should be considered when setting treatment targets and evaluating response to treatment [8]. Recently, we showed that mixed type I (IFNα)- and type II (IFNγ)- interferon response gene signatures in peripheral blood are associated with MTX response [9], whereas International League of Associations for Rheumatology (ILAR) classification criteria in general cannot predict response to treatment [7]. Predicting the response to MTX would be valuable, as it would enable early identification of non-responders who could benefit different or more aggressive treatments.

Previous analyses revealed a significant correlation between the effect sizes for the 51 genes response score and the categorical ACR response [9]. However, standard response categories lack the nuance to capture the diverging parameters of disease activity identified by Shoop-Worrall et al. Therefore, we tested whether baseline peripheral blood IFN response scores could be associated to these longitudinal trajectories rather than isolated clinical outcomes.

## Methods

This study utilised data from CYP recruited into the Childhood Arthritis Response to Medication Study (CHARMS) [9] and integrated into the CLUSTER initiative [10]. Participants were recruited prior to the commencement of methotrexate (MTX) therapy, as detailed in previous studies [11–15].

To be included, patients were required to have active disease at baseline (T1), defined as an active joint count (AJC) > 0 and/or a Juvenile Arthritis Disease Activity Score (JADAS10) > 1. Furthermore, participants had to have complete clinical data at either baseline (T1) or follow-up (T2, 3–11 months after treatment initiation). Disease trajectories were annotated using multivariate group-based trajectory linear modelling (GBTM), using methodology described previously [7].

Trajectory analysis included 699 children from the CHARMS study, while RNAseq data were from 102 children from within this cohort. Interferon (IFN) response scores measured in peripheral mononuclear cells (PBMC) by bulk RNAseq were calculated using a 51-gene signature (51 IGS) and a 5-gene signature (5 IGS), as described [9], based on samples collected prior to methotrexate therapy. PBMC transcriptome data were extracted from the discovery cohort (82 CYP) and the 26-CYP validation cohort of our previous study (Additional Figure S1) [9]. The IFN scores represent the mean expression of the log2 normalised counts of 51 and 5 genes selected in our previous study [9].

To assess representativeness, the sub-cohort of 102 patients with available IFN scores was compared with the larger clinical cohort of 699 patients using the Standardised Mean Difference (SMD, cut-off 0.2). The associations between the identified trajectory groups and IFN response scores were evaluated using the Kruskal–Wallis test. To assess the association between the trajectory groups the 51IGS and 5IGS signature, we fitted nested linear regression models. A base model including age and sex as a covariate was compared to a full model including age, sex and the trajectory group using an Analysis of Variance (ANOVA) F-test. Post-hoc power calculations assessed the trade-off between statistical power and sensitivity. The sensitivity analysis examined how increasing the number of trajectory groups influenced power and the number of children needed to reach reliable results with sufficient power.

## Results

Initial analysis included 699 CYP from the CHARMS dataset in whom clinical data before MTX and at 3–11 months follow-up were available (Additional Fig. 1). A nested cohort of 102 CYP had available PBMC transcriptomic data for the 51-gene and 5-gene IFN response scores (Additional Figure 1). The representativeness of the sub-cohort relative to the full cohort was assessed using a Standardised Mean Difference (SMD) cut-off of 0.2. Imbalances between the sub-cohort of 102 CYP and the larger cohort were observed primarily in ethnicity (Table 1 and Additional Table 1).

**Fig. 1:**
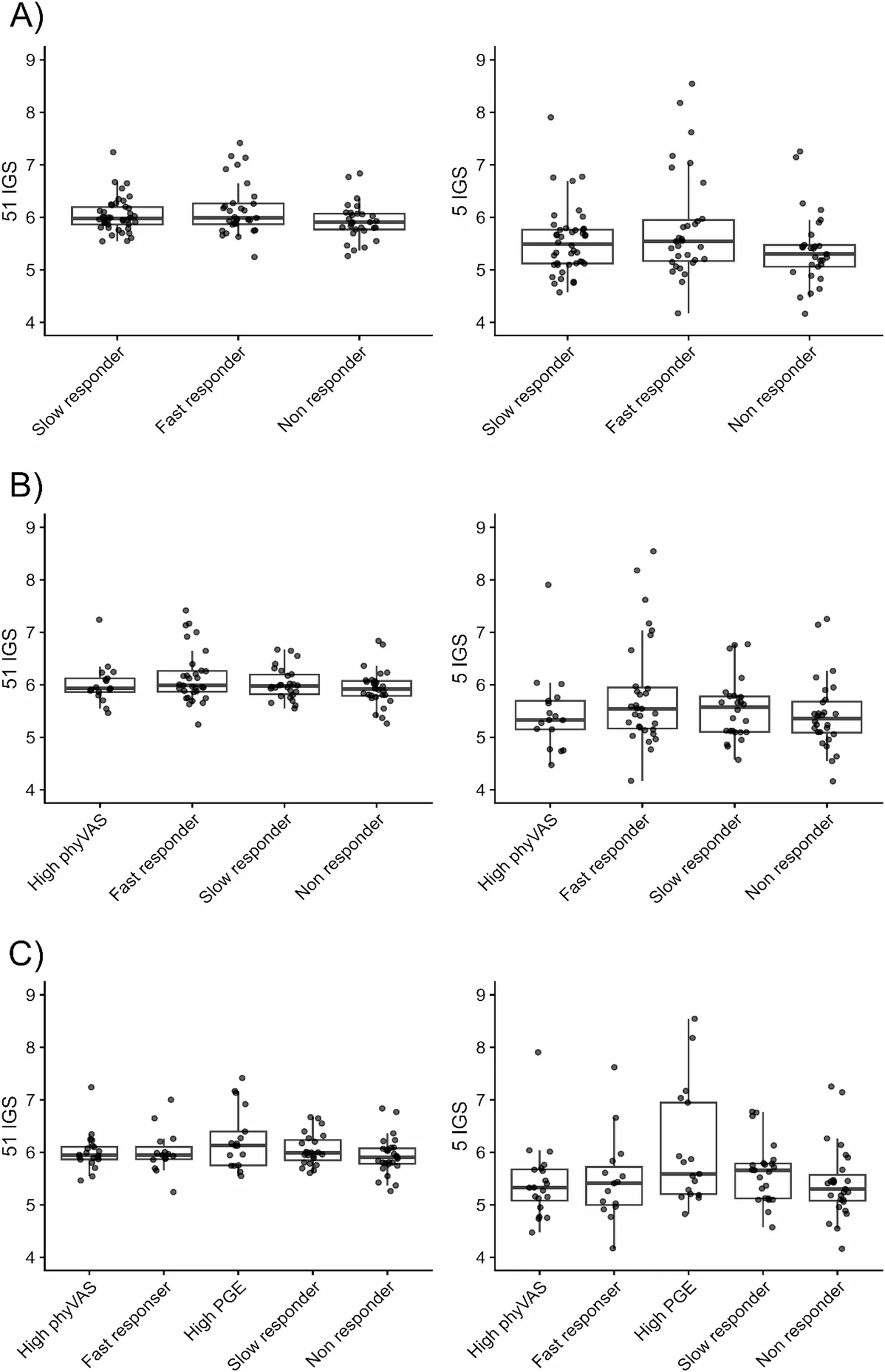
Distribution of the interferon score among machine learning defined classes of response to methotrexate. (**A**) distribution among 3 trajectory classes, (**B**) distribution among 4 trajectory classes (**C**) distribution among 5 trajectory classes. For each of **A**), **B**), **C**) left hand panel shows results with 51-gene score, right hand panel shows results with 5-gene score. SR: Slow Responder group. FR: Fast Responder group. NR: Non-Responder group. High PhyVAS: group with discordance between physician assessment and the other Juvenile Arthritis Disease Score (JADAS) components (active joint count, ESR and parent/patient perception of disease activity). High PGE: group with discordance parent/patient perception of disease activity and the other JADAS components. 51 IGS: 51-gene score. 5 IGS: 5-gene score

**Table 1:** Clinical and demographic characteristics of the children and young people included in this study

	All sample (*N* = 699)		IFN score sample (*N* = 102)		SMD
Female sex, *N* (%)	444	(64.35)	64	(62.75)	0.039
White ethnicity, N (%)	618	(90.62)	68	(73.12)	0.564
MTX dose (mg/m^2^), median (IQR)	12.69	(10.40-14.52)	13.32	(11.65–14.42)	0.201
Age T1 (years), median (IQR)	8.25	(4.17–11.46)	8.03	(4.34–12.35)	0.035
Disease duration T1 (years), median (IQR)	0.74	(0.35–2.18)	0.86	(0.41–2.79)	0.224
ESR T1 (mm/h), median (IQR)	28	(10–58)	21	(8–55)	0.092
CRP T1 (mg/L), median (IQR)	7	(5-25.5)	5	(5-13.75)	0.263
Patient or parent global VAS (0.0–10.0) T1, (median, IQR)	4.1	(1.8–6.5)	3.95	(1.45–5.97)	0.118
Active joints T1, median (IQR)	6	(3–10)	4.5	(3–8)	0.252
Physician global disease activity VAS (0.0–10.0) T1, median (IQR)	4	(2.5-6.0)	4.05	(2.3–5.75)	0.05
ESR T2 (mm/h), median (IQR)	10	(5–24)	12	(5-20.25)	0.073
CRP T2 (mg/L), median (IQR)	5	(2.0–9.0)	5	(4-0-9.25)	0.171
Patient or parent global VAS (0.0–10.0) T2, (median, IQR)	1.5	(0.1-4.0)	1.1	(0.0-3.3)	0.225
Active joints T2, median (IQR)	1	(0–4)	0	(0–2)	0.337
Physician global disease activity VAS (0–10.0) T2, median (IQR)	1.4	(0.2-3.0)	1	(0.0-2.42)	0.222

CRP: C-reactive protein. ESR: erythrosedimentation rate. IQ: interquartile range. MTX: methotrexate. SMD: Standardised Mean Difference. T1: baseline. T2: timepoint after methotrexate therapy comprised between 3 and 11 months. VAS: Visual Analogue Scale from 0.0 to 10.0 cm

Trajectory models were tested to capture the heterogeneous response to methotrexate. Model selection was guided by the Bayesian Information Criterion (BIC) alongside clinical interpretability (Additional Fig. 2), as explored previously [7]. We rejected any models containing clusters with fewer than 5% of the cohort to ensure the results remained clinically meaningful, and we obtained three optimal models for 3, 4, 5 groups. The JADAS components had distinct trends that allowed to identify clinically meaningful trajectory groups (Additional Table 2 and Additional Figs. 3, 4 and 5). Sensitivity analyses confirmed that neither ethnicity nor baseline MTX dose significantly correlated with trajectory group assignment or IFN response scores.

Among the 102 individuals with transcriptomic data, a strong correlation was observed between the 51 IGS (range 5.2–7.4) and the 5 IGS (range 4.2–8.5) (Pearson’s *r* = 0.93, p-value < 0.001, Additional Fig. 6).

The 51- gene and 5- gene IFN scores were compared across the 3, 4, and 5 trajectory groups (Fig. 1). Kruskal-Wallis test revealed no statistically significant differences in score distributions for either the 51 IGS (3 groups p-value = 0.180; 4 groups p-value = 0.506; 5 groups p-value = 0.478) or the 5 IGS (3 groups p-value = 0.232; 4 groups p-value = 0.513; 5 groups p-value = 0.207). After adjusting for age and sex, the inclusion of trajectory groups in the linear model did not significantly improve the prediction of IFN scores (Additional Table 3). This indicates that trajectory group membership did not independently account for the variance observed in the IFN signature.

The post-hoc power calculation found that our study had limited power (44%) to detect medium effect sizes due to the limited size of the smallest cluster (*n* = 12). Indeed, we found that only large differences (Cohen’s f = 0.36) between trajectory groups could be detected with the available sample and that we would need a mean 39 individuals per trajectory group instead of our 18 (harmonic mean). Power rises slightly when considering 4-trajectory groups (50%) or 3-trajectory groups (58%), however Cohen’s f values do not decrease significantly (4 groups f = 0.31; 3 groups f = 0.34) (Additional Fig. 7). Our post-hoc power calculations demonstrated an unfavourable trade-off: as the number of trajectory classes rises to better capture clinical nuance, statistical power wanes, whereas sensitivity remains low. Conversely, reducing the number of classes increases power but results in simplistic models (dichotomising outcomes into 2 or 3 classes) that fail to capture the true variety of the MTX response.

## Discussion

The results of our study confirm that response to MTX is heterogeneous and difficult to capture by categorical classifications. The primary contribution of this work is the shift from evaluating biomarkers against uni-dimensional outcomes at one time point to testing their predictive utility across multivariate trajectory classes. Univariate continuous measures such as change in active joint count or JADAS are more powerful outcomes for statistical tests, but categorical trajectories are needed to describe the longitudinal response patterns across multiple dimensions. However, statistical power tends to be limited by such grouping of individuals, because information about contrasts between individuals within a group cannot be exploited by statistical tests. Further, while using more groups can recapture some of these contrast information by subdividing groups, the statistical test needs to use more degrees of freedom, further reducing power. While the trajectory modelling identified nuanced groups, baseline IFN signatures could not distinguish between them in this study. Crucially, the absence of a statistically significant association in this cohort should not be misinterpreted as definitive evidence of no biological relationship. Determining whether associations of baseline IFN with single continuous response outcomes extend to multidimensional trajectories will require larger cohorts, potentially multi-centre international cohorts.

The discrepancy between the IFN signature’s ability to predict overall MTX improvement versus its failure to predict specific trajectory classes may stem from the multi-dimensional nature of these trajectories. Clinical response to MTX may be driven not only by the biological pathways captured by interferon signalling, but also by drug intolerance, psychosocial factors, pain perception, and mechanical damage. In this context, the five-group trajectory model may distinguish nuanced response patterns that account for these non-biological drivers. For instance, the “High PGE” group reflects a phenotype where children show objective improvement (reduced joint count, ESR, and Physician VAS) but do not report improved wellbeing, potentially due to MTX intolerance.

Despite the lack of predictive power observed in this study, the clinical imperative for early stratification remains acute. Current management often relies on a “step-up” approach, where biologic agents are introduced only after MTX inefficacy [3]. However, evidence increasingly supports the existence of a “window of biological opportunity,” where the early introduction of effective therapy—including biologics—is associated with significantly higher chances of achieving inactive disease within 6 months [16]. The inability to identify MTX nonresponders at diagnosis results in a “wait-to-fail” paradigm, potentially delaying effective treatment during this critical window. Therefore, while peripheral blood IFN scores alone may not suffice as a stratification tool for these specific trajectories, the integration of biomarkers remains a priority to facilitate a treat-to-target strategy that is personalized from onset [10].

## Supplementary Information

Below is the link to the electronic supplementary material.

Supplementary Material 1

## Data Availability

No datasets were generated or analysed during the current study.

## Abbreviations

JIA: Juvenile Idiopathic Arthritis
IFN: Interferon
MTX: Methotrexate
51 IGS: 51 interferon genes signature
5 IGS: 5 interferon gene signature
PGE: Patient or parent global assessment
PhyVAS: Physician global assessment of disease activity
PBMC: Peripheral blood mononuclear cells
CYP: Children and young people
SMD: Standardised Mean Difference
AJC: Active joint count
JADAS: Juvenile Arthritis Disease Activity Score
GBTM: Group-based trajectory linear modelling
DMARDs: Disease-modifying anti-rheumatic drugs
ESR: Erythrosedimentation rate
