# Supplementary material for: Challenges in linking interferon signatures to clinical trajectories in juvenile idiopathic arthritis
Source: Pediatr Rheumatol Online J. 2026 Aug 18;24:86. doi: 10.1186/s12969-026-01256-x (PMC13760786; doi:10.1186/s12969-026-01256-x)
Supplement: Supplementary file 1 [file 12969_2026_1256_MOESM1_ESM.docx]

**Scagnellato et al 2026**

**Additional material**

**Additional Table 1. Missing values.** Counts and percentages of missing data in the overall cohort and the subset.

| Variable | Whole cohort (N = 699)  Missing counts (%) | | Subset (N = 102)  Missing counts (%) | |
| --- | --- | --- | --- | --- |
| Female sex | 9 | (1.29) | 0 | (0) |
| White ethnicity | 17 | (2.43) | 9 | (8.82) |
| Follow-up, months | 52 | (7.44) | 23 | (22.55) |
| Disease duration T1 (years) | 72 | (10.3) | 34 | (33.33) |
| MTX dose (mg/m^2^) | 98 | (14.02) | 35 | (34.31) |
| Age T1 (years) | 12 | (1.72) | 0 | (0) |
| Age of onset (years) | 39 | (5.58) | 9 | (8.82) |
| ESR T1 (mm/h) | 44 | (6.29) | 3 | (2.94) |
| CRP T1 (mg/L) | 264 | (37.77) | 12 | (11.76) |
| Patient or parent global VAS (0-10) T1 | 120 | (17.17) | 4 | (3.92) |
| Active joints T1 | 16 | (2.29) | 0 | (0) |
| Physician global disease activity VAS (0-10) T1 | 81 | (11.59) | 0 | (0) |
| ESR T2 (mm/h) | 120 | (17.17) | 18 | (17.65) |
| CRP T2 (mg/L) | 364 | (52.07) | 34 | (33.33) |
| Patient or parent global VAS (0-10) T2 | 93 | (13.3) | 3 | (2.94) |
| Active joints T2 | 37 | (5.29) | 0 | (0) |
| Physician global disease activity VAS (0-10) T2 | 74 | (10.59) | 0 | (0) |

CRP: C-reactive protein. ESR: erythro-sedimentation rate. MTX: methotrexate. SMD: Standardised Mean Difference. T1: baseline. T2: timepoint after methotrexate therapy comprised between 3 and 11 months. VAS: Visual Analogue Scale from 0 to 10 cm.

**Additional Table 2.** Distribution of 699 CYP among the trajectory groups according to estimated posterior probabilities in three GBTM models.

|  | Model for 3 groups | Model for 4 groups | Model for 5 groups |
| --- | --- | --- | --- |
| High PGE | / | / | 12.1% |
| Fast responders | 30.4% | 30.4% | 12.4% |
| High phyVAS | / | 16.7% | 11.9% |
| Slow improvers | 41.2% | 25.5% | 26.9% |
| Non-responders | 28.4% | 27.5% | 37.0% |

**Additional Table 3. Outputs of the linear models adjusting for age and sex.**

| Trajectory groups | 51-ISG (p-value) | 5 -ISG (p-value) |
| --- | --- | --- |
| 3 groups trajectory | 0.103 | 0.103 |
| 4 groups trajectory | 0.281 | 0.281 |
| 5 groups trajectory | 0.1992 | 0.1992 |

**Additional Figure 1. Flowchart of the included children and young people (CYP).** We identified 1211 potential CYP initiating methotrexate (MTX) within the CHARMS cohort (1). Among these, 108 CYP had transcriptomic data (2). After applying inclusion and exclusion criteria, 512 individuals were removed from the analysis. This yielded a final dataset of 699 CYP for trajectory annotation and 102 individuals with transcriptomic data for the interferon score annotation. JADAS: Juvenile Arthritis Disease Activity Score; T1: baseline, time of MTX initiation; T2: second assessment after MTX initiation; AJC: active joint count.

**Additional Figure 2.** **Output of the multivariate group-based trajectory linear modelling (GBTM)** to classify 699 children into distinct trajectory groups. The optimal models were the 3 groups, 4 groups and 5 groups models.

**Additional Figure 3. JADAS components trends over time in the three trajectory groups distribution.** Trajectories were named based on the trends of the four JADAS components and annotated by colour. JADAS: Juvenile Arthritis Disease Activity Score, including ; AJC: active joint count; phyVAS: physician global disease assessment on Visual Analogue Scale 0-10 cm; PGE: patient or parent global health assessment on Visual Analogue Scale 0-10 cm; ESR: erythron-sedimentation rate (mm/h).

**Additional Figure 4. JADAS components trends over time in the four trajectory groups distribution.** Trajectories were named based on the trends of the four components and the previous validation in (1). Groups are annotated by colour. JADAS: Juvenile Arthritis Disease Activity Score, including AJC: active joint count; phyVAS: physician global disease assessment on Visual Analogue Scale 0-10 cm; PGE: patient or parent global health assessment on Visual Analogue Scale 0-10 cm; ESR: erythron-sedimentation rate (mm/h).

**Additional Figure 5. JADAS components trends over time in the five trajectory groups distribution.** Trajectories were named based on the trends of the four components and the previous validation in (1). Colour indicates the trajectory group. JADAS: Juvenile Arthritis Disease Activity Score, including AJC: active joint count; phyVAS: physician global disease assessment on Visual Analogue Scale 0-10 cm; PGE: patient or parent global health assessment on Visual Analogue Scale 0-10 cm; ESR: erythron-sedimentation rate (mm/h).

**Additional Figure 6. Interferon scores distribution and correlation.** The 51 genes (A) and the 5 genes (B) interferon score are not normally distributed. The 51 genes and the 5 genes interferon scores are strongly linearly correlated (C). 51 IGS: 51 genes interferon score; 5 IGS: 5 genes interferon scores.

**Additional Figure 7. Power curves showing that power decreases while increasing**

**the number of trajectory groups.** The dashed lines identify the target power and the target

effect.

References

1.	Shoop-Worrall SJW, Lawson-Tovey S, Wedderburn LR, Hyrich KL, Geifman N, Consortium C. Towards stratified treatment of JIA: machine learning identifies subtypes in response to methotrexate from four UK cohorts. EBioMedicine. 2024;100:104946.

2.	Kartawinata M, Lin WY, Jebson B, O'Brien K, Ralph E, Welsh E, et al. Identification and validation of interferon-driven gene signature as a predictor of response to methotrexate in juvenile idiopathic arthritis. Ann Rheum Dis. 2025;84(8):1412–24.
